# Patterns of Self-Care Behaviors and Their Influence on Maintaining Independence: The National Health and Aging Trends Study

**DOI:** 10.3389/fragi.2021.770476

**Published:** 2021-12-24

**Authors:** Thelma J. Mielenz, Sneha Kannoth, Qian-Li Xue

**Affiliations:** ^1^ Department of Epidemiology, Mailman School of Public Health, Columbia University, New York, NY, United States; ^2^ Division of Geriatric Medicine and Gerontology, School of Medicine Department of Medicine and the Center on Aging and Health, The Johns Hopkins University, Baltimore, MD, United States

**Keywords:** physical activity, sleep, patterns of behavior, community engagement, mortality

## Abstract

**Importance:** Few studies have addressed the combined effects of health-promoting and self-care behaviors among older adults. Thus, new research is needed to assess the potential for behavior change to prolong independence in later life.

**Objectives:** To determine the relationships between self-care behaviors and risks of mobility and activities of daily living (ADLs) over time.

**Design:** Longitudinal data was used from the National Health and Aging Trends Study (NHATS) cohort. Eight baseline self-care behaviors were summarized using latent class analysis. Separately, longitudinal latent classes of mobility and ADLs were created.

**Setting:** Annual in-person interviews conducted for a nationally representative sample.

**Participants:** The baseline study sample included 7,609 Medicare beneficiaries aged ≥65 from NHATS who were living in community or residential care settings, with a 71% response rate. The average age was 75, with 57% female, 81% white and 78% high school graduates or higher. Approximately, 80% (n = 6,064) completed 5 years of follow-up.

**Exposures:** Favorable vs. unfavorable self-care latent classes measured at baseline.

**Main outcomes and Measures:** Associations were measured between baseline classes and longitudinal classes of mobility and ADLs difficulty. Among decedents, 5-year associations were measured between baseline classes and years of overall, healthy, able, and healthy/able life.

**Results:** Two habitual baseline self-care behavioral patterns (46% favorable; 54% unfavorable) and three trajectories of change in mobility and ADLs disability (maintaining independence; shifting to accommodation/difficulty; shifting to assistance) emerged over time. Participants with a favorable baseline pattern had 92% (0.90–0.94) reduced risk in shifting to assistance class and 70% (0.64–0.76) reduced risk for shifting to accommodation/difficulty class for mobility disability. Participants with a favorable baseline pattern had 86% (0.83–0.89) reduced risk in shifting to assistance class and 24% (0.11–0.36) reduced risk in shifting to accommodation/difficulty class for ADLs disability. Those with an unfavorable pattern had 2.54 times greater risk of mortality by the end of the 5-year follow-up compared to those with a favorable pattern.

**Conclusion:** Self-care behaviors in older age represent a habitual pattern. A favorable self-care behavioral pattern decreased the risk of moving towards a more disabled profile and added years of life. Interventions should encourage self-care behaviors constituting a favorable pattern.

## Highlights

### Question

What are the self-care behaviors associated with independence in older age?

### Findings

Analyses of the National Health and Aging Trends cohort (2011–2016) demonstrated that for mobility and activities of daily living disability outcomes respectively, older adults with a favorable self-care behavioral pattern had a 92 and 87% reduced risk in shifting to the assistance class, along with a 71 and 27% reduced risk in shifting to the accommodation/difficulty class compared to those with an unfavorable self-care pattern.

### Meaning

Comprehensive interventions must focus on shifting an unfavorable self-care pattern to a favorable self-care pattern among older adults.

## Introduction

Healthy aging has been defined as “the process of developing and maintaining functional ability that enables well-being in older age.” (Michel and Sadana, 2017) Self-care encompasses behaviors that individuals may engage in to improve their health, well-being, and functioning as they become older (Levin and Idler, 1983) Examples of self-care may include healthy eating, sleep, financial fitness, healthy relationships, medication management, exercise, community engagement and falls prevention (Aging Mastery Program, 2020) Given that self-care practices are modifiable, individuals can maximize their health benefits. Existing studies support the proposition that even the oldest members of society can make important gains in health by initiating self-care physical activity programs (Fiatarone et al., 1994) Sleep problems affect activities of daily living and functional impairment as well as cognitive and mental health not to mention chronic disease (Vanderlinden et al., 2020) Self-care behaviors may address the improvement of cognitive function among older adults but this paper will focus on the physical functioning.

To gauge the impact of these health gains achieved through self-care, researchers have examined the influence of lifestyle factors on the compression or expansion of the disabled period late in life. Years of able life were used to define a period during which individuals experienced no difficulties with activities of daily living (ADLs) (Jacob et al., 2016) In addition to evaluating years of able life, mortality rates may also serve as a metric to ascertain health benefits of self-care. Previous research suggests that declines in mortality are greatest among those maintaining physical activity, and even low-intensity programs can contribute to longevity (Xue et al., 2012) Nevertheless, there is much to learn about how changes across a range of healthy behaviors in older adults might prolong independence.

Using a framework for studying patterns of change in longitudinal physical activity behaviors and mortality risk in older women (Xue et al., 2012), we used the nationally representative National Health and Aging Trends Study (NHATS) to: 1) identify subpopulations among Medicare beneficiaries 65 and older with distinct baseline patterns of self-care behaviors, 2) to examine the relationship of baseline individual self-care behavior patterns on mobility and ADLs disability over time, and 3) to explore how these self-care behavioral patterns influence the years of healthy and able life. We hypothesize that healthy self-care behaviors can lower risk for functional dependence over time and that there is a differential risk decrease depending on the self-care behavior patterns. We also hypothesize that self-care behavioral patterns can compress the number of disabled years.

## Materials and Methods

### Study Sample

The NHATS cohort is a nationally representative sample of Medicare beneficiaries aged 65 years and older (Kasper and Freedman, 2015) NHATS study aims include understanding trends in functioning and disability as individuals age. NHATS contains extensive information on self-care behavior, and functioning across a range of routine daily activities. Data are collected annually through in-person interviews; the first six annual rounds (2011–2016) were used for this analysis. The response rate was 71% at baseline (n = 8,245). Older adults who were living in community-dwelling or residential care settings (n = 7,609) were included in this study.

### Predictor

#### Self-Care Behaviors

Measures of self-care behaviors include 1-low physical activity, 2-insomnia symptoms, 3-financial stability, 4-healthy relationships, 5-community engagement, 6-medication management, 7-non-physical activities, and 8-interaction with email/text and the internet. We describe each of these in detail (Table 1).

1. Low physical activity was defined using questions regarding walking for exercise and engaging in vigorous activity in the last month. Individuals were classified as doing neither, one, or both (Bandeen-Roche et al., 2015).
2. Poor sleep quality or insomnia symptoms were defined using two items (Endeshaw and Yoo, 2016): “How often does it take you more than 30 min to fall asleep at night?” and “How often do you have trouble falling back to sleep on nights after waking up from sleep?” We categorized those who endorsed any frequency of either insomnia symptom as positive and created four categories: 1-no insomnia symptoms, 2-difficulty initiating sleep only, 3-difficulty maintaining sleep only, and 4-both insomnia symptoms.
3. Financial stability was defined using several items. A score of one was assigned for: paying off credit card balances or not having cards, not receiving financial help from family, giving financial gifts/help to family, not receiving food stamps or food assistance or gas or energy assistance. A summary score was created (0–4); higher scores indicate greater economic stability (Freedman et al., 2011).
4. Healthy relationships were defined by self-report of an in-person visit with outside family or friends and whether health or functioning ever prevented visits with outside family and friends. A four-category variable was created: 0-no visit and without restriction, 1-no visit due to restriction, 2-visit despite restriction, and 3–visit and without restriction (Latham and Clarke, 2018).
5. Community engagement was defined using several items. A score of one was assigned for: attending religious services; participating in clubs, classes or other organized activities; going out for enjoyment; and doing volunteer work. A summary score was created (0–8); higher scores indicate greater engagement. Participants also self-reported whether or not health or functioning prevented community engagement.
6. Medication management was categorized in three categories: someone else manages medications, person manages own medications with difficulty, and person manages own medications without difficulty or takes no medications.
7. Self-report of an individual’s favorite activity was grouped into physical or non-physical activities based on the amount of mobility or strength required (Szanton et al., 2015). A dichotomous variable was then used for self-report of non-physical activities. For round 6, we carried forward the round five favorite activity.
8. Interaction with technology was defined by self-reporting use of e-mail/text, and use of internet. A three-level variable (doing neither, one, or both) was created (Gell et al., 2015).

**TABLE 1:** Summary of baseline characteristics by self-care behavioral patterns^a^.

Baseline characteristic % or mean (standard error)	Total sample (n = 7,609)	Unfavorable behavioral pattern (n = 4,128; 47.3%)	Favorable behavioral pattern (n = 3,481; 52.7%)	*p*-value^a^
Age (year)	75.3 (0.09)	77.1 (0.13)	73.7 (0.11)	<0.001
Race				
White, non-Hispanic	80.5%	73.8%	86.6%	<0.001
Black, non-Hispanic	8.1%	11.0%	5.5%	
Hispanic	6.7%	9.7%	4.0%	
Other	4.6%	5.4%	3.9%	
Sex (Female)	56.6%	60.7%	52.9%	<0.001
Education				
≤8th grade	10.4%	17.4%	4.1%	<0.001
9th-12th grade (no diploma)	11.4%	17.0%	6.3%	
High school graduate or higher	78.2%	65.6%	89.6%	
Physical activity (walk, vigorous activities)				
Neither	30.5%	55.9%	7.7%	<0.001
Either	39.0%	39.4%	38.6%	
Both	30.6%	4.8%	53.8%	
Sleep quality				
no insomnia	72.9%	60.0%	84.5%	<0.001
difficulty initiating sleep only	11.5%	15.9%	7.5%	
difficulty maintaining sleep only	5.5%	6.8%	4.3%	
both insomnia symptoms	10.1%	17.3%	3.7%	
Financial stability (0–4)	2.94 (0.01)	2.66 (0.02)	3.19 (0.01)	<0.001
Healthy relationships				
no visit, no restriction	10.0%	18.4%	2.5%	<0.001
no visit, with restriction	2.5%	5.3%	0.0%	
visit, with restriction	6.6%	12.7%	1.0%	
visit, no restriction	80.9%	63.6%	96.4%	
Community Engagement (0–8)	4.22 (0.03)	3.09 (0.03)	5.23 (0.04)	<0.001
Medication Management				
Self-manage, no difficulty/no meds	85.9%	76.9%	93.9%	<0.001
Self-manage, difficulty	7.1%	8.2%	6.1%	
Others manage	7.0%	14.8%	0%	
Non-physical activities				
Yes	35.6%	47.9%	24.6%	<0.001
Email/text and internet				
Both email/text and internet	36.5%	8.9%	57.1%	<0.001
Either email/text or internet	17.6%	15.9%	18.9%	
Neither	45.9%	75.2%	24.0%	

a Weighted percentages to ensure population representation.

### Outcomes

#### ADL and Mobility Scale

We developed two scales in order to assess the risk for functional dependence adapted from a late-life hierarchical disability scale developed by Freedman et al. (Freedman et al., 2014; Gill and Williams, 2017) One scale assessed activities of daily living or ADLs (using the toilet, getting cleaned up, dressing, and eating) and the other measured mobility activities (getting out of bed, going outside and getting around inside). Each scale ranged from: “fully able (0: no device use, reduction in activities, difficulty, or assistance on any activity); accommodations or reductions in activities (1: device use or reductions in one or more activities but no difficulty or assistance); difficulty (2: difficulty performing activities by oneself, when using devices, if needed, but no assistance); and assistance (3: help from another person or, rarely, not doing the particular activity).” (Freedman et al., 2014; Gill and Williams, 2017).

### Years of Able Life and Healthy Life

Measures of remaining lifespan and health span were created among the 1,575 study subjects who died during the 5-year follow-up by summing the number of years when a person was observed to be alive (i.e., Years of Life; YOL), healthy (i.e., Years of Healthy Life; YHL), able (i.e., Years of Able Life; YABL), and both healthy and able (i.e., Years of Healthy and Able Life: YHAL). We defined “healthy” as self-rated health of being excellent, very good, or good (as opposed to fair or poor health); we defined “Able” as self-report of neither having difficulty nor needing assistance in performing ADLs or mobility tasks; and we defined “Healthy and Able” as meeting both criteria. We used the last observation carried forward to impute intermittent missing data on self-rated health and disability status.

### Covariates

Sex, age (years), race (non-Hispanic white, non-Hispanic black, Hispanic and other), and education level (eighth grade or less, 9th-12th grade without high school diploma, and high school or greater).

### Statistical Analysis

All analyses were weighted for population representation using replicate weights that employ the modified balanced repeated replication method to adjust for the complex survey design of NHATS (DeMatteis et al., 2016) First, adoption of individual self-care behaviors were examined at baseline and at each follow-up visit. Subpopulations that exhibit homogeneous self-care behavioral patterns were identified using a latent class model (LCA) (Bandeen-Roche et al., 1997) The LCA was fit in two ways: 1) analysis of patterns of coexistence of the eight self-care behaviors at baseline, and 2) analysis of patterns of change in each self-care behavior over time. Using the former as an example, the LCA hypothesizes the existence of subpopulations of older adults with distinct self-care behavior patterns at baseline.

The LCA then aims to determine the number of subpopulations (“behavior patterns”) and estimates for (i) its prevalence in the overall population, and (ii) the proportions (i.e. item probabilities) who were at different levels for each behavior at baseline within each subpopulation. The number of subpopulations is based on the model goodness-of-fit statistics including Akaike information criterion (AIC), Bayesian information criterion (BIC), Lo-Mendell-Rubin adjusted likelihood ratio test, model performance stability, and the scientific plausibility and meaningfulness of the resulting behavioral patterns (Lo et al., 2001) To account for missing data, the LCA model is fit using the full information maximum likelihood estimator that is unbiased under the assumption of data missing at random (Rubin, 1976)

Upon completing the LCA, we assigned individuals to specific behavioral pattern profiles corresponding to the highest posterior probability of latent class membership. Next, we applied the LCA to study within-subject change in self-care behaviors over time by running separate LCA for each self-care behavior; similarly, we ran separate LCA for modeling changes in the level of difficulty in mobility and ADLs disability over time. We also modeled the association between baseline self-care behavioral patterns and change in mobility and ADLs disability over time using latent class regression (LCR). The LCR characterizes the impact of self-care behaviors on the trajectory of late-life disability, while accounting for within-person dependence over time in individual trajectories as well as data missing at random (Rubin, 1976) Lastly, we assessed how our baseline self-care behavioral patterns were associated with YOL, YAL and YHL, and YHAL (Fiatarone et al., 1994) To do so, we used Poisson regression for YoL, and negative binomial regression for YAL, YHL, and YHAL to account for excess zeros (i.e., those who reported poor or fair health and/or difficulty or needing assistance with ADLs or mobility tasks consistently throughout the follow-up period) and sampling weights were incorporated in the estimation of the ratio for population representation. In addition, we analyzed the association between baseline self-care behavioral patterns and all-cause mortality among all those who died. Analyses were conducted using Stata 15 (StataCorp, 2015) and Mplus 6.1.1 (Muthén and Muthén, 2010).

## Results

The average age of the study sample was 75, with 57% female, 81% Non-Hispanic white, and 78% high school graduates or higher. About one-third of the population was sedentary; 10% reported poor sleep quality; 13% had limited social networks, and 50% were technology-averse (Table 1).

### Self-Care Behavior Patterns at Baseline and Over Time

There was little variation in self-care behaviors over time (Supplementary Table 1, 6A–F), suggesting that behavioral patterns observed at older ages may represent long-term habitual patterns. Two baseline self-care behavioral patterns emerged from the LCA, favorable and unfavorable. The two-class model was selected based on lower BICs and the significance of the Lo-Mendell-Rubin adjusted likelihood ratio test (LMR-LRT), with the number of latent classes chosen to be one class less than the number corresponding to the model with non-significant LMR-LRT (Supplementary Table 3). Compared to an unfavorable pattern at baseline, a favorable pattern had more: physical activity (54 vs. 5%), sleep quality (85 vs. 60%), financial stability (3.2 vs. 2.7), healthy relationships (96 vs. 64%), community engagement (5.2 vs. 3.1), email, text and internet use (57 vs. 9%), less medication management (0 vs. 15%), and less non-physical activities (25 vs. 48%) (Table 1). Additionally, those exhibiting a favorable pattern were likely to be younger (74 vs. 77), Non-Hispanic white (87 vs. 74%), and more educated (90 vs. 66% with high school diploma or higher).

### Mobility and ADL Disability Trajectory Profile Over Time

**FIGURE 1:**
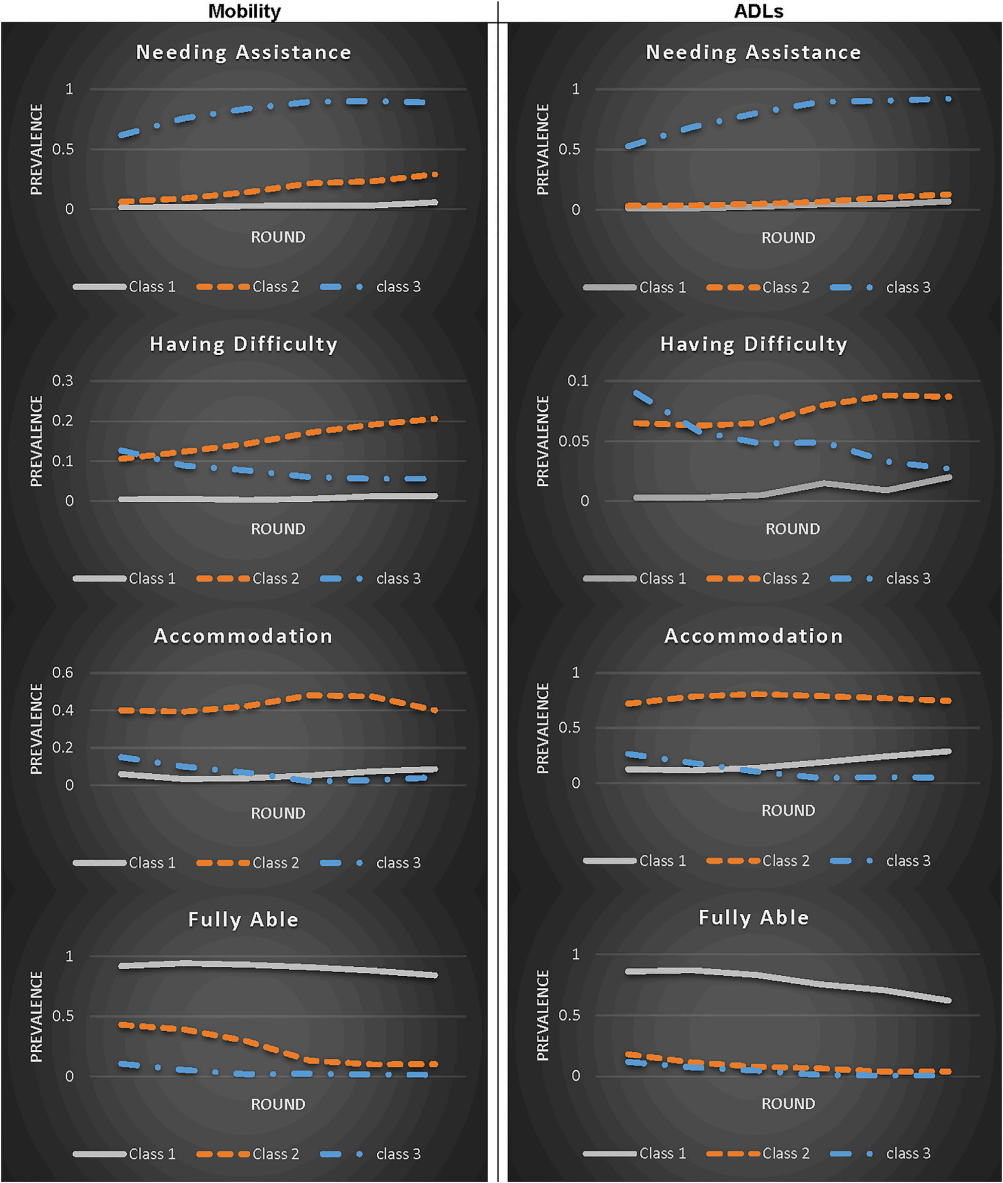
Mobility and activities of daily living (ADLs) disability trajectory profiles derived from a 3-Class latent class model (class 1: maintaining independence over time; Class 2: shifting to accommodation or difficulty; Class 3: shifting to assistance).

A three-class model was chosen to represent trajectory profiles for both mobility and ADLs (Table 2; Figure 1). The classes were named: Class 1-maintaining independence over time, Class 2-shifting to accommodation or difficulty, and Class 3-shifting to assistance. Older age, lower education, and non-white race were also associated with greater risk of developing difficulty and dependency in mobility and self-care tasks (Table 2).

**TABLE 2:** Summary of baseline characteristics by mobility and activities of daily living (ADLs) disability trajectory profile over time (Class 1: maintaining independence over time; Class 2: shifting to accommodation or difficulty; Class 3: shifting to assistance).

Variable % or mean (s.e.)	Mobility			ADLs		
	Class 1	Class 2	Class 3	Class 1	Class 2	Class 3
	n = 4,132	n = 1,648	n = 1,829	n = 2,507	n = 3,158	n = 1,944
Age (year; n = 7,609)	73.0 (0.1)	77.4 (0.2)	80.9 (0.2)	72.3 (0.1)	75.7 (0.1)	80.1 (0.2)
Sex (female)	50.9%	63.4%	69.2%	44.9%	64.2%	63.5%
Race (n = 7,609)						
White, non-Hispanic	83.1%	77.9%	74.5%	78.3%	84.6%	76.4%
Black, non-Hispanic	6.5%	11.3%	10.4%	8.0%	7.3%	10.1%
Hispanic	5.6%	6.8%	10.6%	7.9%	4.4%	9.3%
Other	4.8%	4.0%	4.5%	5.8%	3.7%	4.1%
Education (n = 7,513)						
≤8th grade	6.8%	13.1%	20.0%	9.6%	7.5%	18.1%
9th-12th grade (no diploma)	9.9%	12.2%	15.5%	10.3%	10.9%	14.5%
High school graduate or higher	83.3%	74.7%	64.6%	80.0%	81.7%	67.4%
Physical activity (walk, vigorous activities; n = 7,602)						
Neither	20.7%	36.2%	58.2%	19.5%	29.4%	53.7%
Either	39.1%	43.9%	33.5%	38.3%	41.2%	35.4%
Both	40.3%	19.9%	8.3%	42.1%	29.4%	10.9%
Sleep quality (n = 7,564)						
no insomnia	78.6%	65.9%	60.7%	77.7%	73.3%	62.9%
difficulty initiating sleep only	9.8%	13.7%	15.0%	10.2%	11.5%	13.9%
difficulty maintaining sleep only	5.0%	6.7%	6.0%	5.3%	5.3%	6.1%
both insomnia symptoms	6.7%	13.8%	18.3%	6.8%	9.8%	17.2%
Financial fitness (0–4; n = 6,830)	3.02 (0.01)	2.85 (0.03)	2.74 (0.02)	3.00 (0.02)	2.95 (0.02)	2.78 (0.02)
Healthy relationships (n = 7,598)						
no visit, no restriction	7.8%	13.9%	13.5%	9.4%	9.1%	13.1%
no visit, with restriction	0.4%	2.1%	10.5%	0.4%	1.3%	9.2%
visit, with restriction	2.5%	9.0%	18.0%	2.8%	5.6%	15.9%
visit, no restriction	89.3%	75.0%	58.0%	87.4%	84.1%	61.8%
Community engagement (0–8; n = 7,583)	4.46 (0.04)	4.02 (0.06)	3.57 (0.05)	4.24 (0.05)	4.45 (0.04)	3.70 (0.05)
Medication management (n = 7,609)						
Self-manage, no difficulty/no meds	93.3%	86.3%	60.0%	94.1%	89.8%	62.1%
Self-manage, difficulty	5.6%	9.2%	10.2%	4.7%	8.2%	9.4%
Others manage	1.1%	4.6%	29.8%	1.2%	2.0%	28.6%
Non-physical activities (n = 7,609)						
Yes	27.9%	44.4%	53.1%	27.1%	37.4%	48.4%
Internet/email use						
Both email/text and internet	43.9%	25.2%	14.1%	43.8%	37.0%	15.5%
Either email/text or internet	18.9%	17.4%	11.4%	17.7%	19.0%	12.6%
Neither	37.2%	57.4%	74.5%	38.5%	44.0%	70.9%

### Baseline Self-Care Behavior Patterns and Patterns of Change in Mobility and ADL Disability Over Time

There were stepwise associations between behavioral and disability outcomes with more favorable self-care behavior patterns (i.e., more physical activity, better sleep, greater community engagement) associated with greater likelihood of maintaining independence over time and decreased likelihood of becoming dependent. Participants with a favorable baseline self-care behavioral pattern had a 92% (95% CI:0.90–0.94) reduced risk in shifting to the assistance class and a 70% (95% CI:0.64–0.76) reduced risk of shifting to the accommodation/difficulty class for mobility disability than those with an unfavorable pattern (Table 3) over 5 years. Those with a favorable self-care behavioral pattern had an 86% (95% CI:0.83–0.89) reduced risk of shifting to the assistance class and a 24% (95% CI:0.11–0.36) reduced risk of shifting to the accommodation/difficulty class for ADLs disability than those with an unfavorable pattern over 5 years (Table 4).

**TABLE 3:** Associations of individual self-care behavior or self-care behavior patterns at baseline and patterns of change in mobility disability over time: Results from latent class regression analysis after adjusting for age, sex, race, and education.

Self-care behaviors or patterns	Shifting to assistance vs. maintaining independence			Shifting to accommodation/difficulty vs. maintaining independence		
	Odds Ratio	95% CI	*p*-value	Odds Ratio	95% CI	*p*-value
Favorable vs. unfavorable behavioral pattern	0.08	(0.06, 0.10)	<0.001	0.30	(0.24, 0.36)	<0.00
Physical activity (walk, vigorous activities)						
Neither	1	-	-	1	-	-
Either	0.60	(0.49, 0.74)	<0.001	0.25	(0.20, 0.30)	<0.001
Both	0.26	(0.21, 0.33)	<0.001	0.07	(0.05, 0.09)	<0.001
Sleep quality						
no insomnia	1	-	-	1	-	-
difficulty initiating sleep only	1.94	(1.50, 2.50)	<0.001	2.36	(1.79, 3.10)	<0.000
difficulty maintaining sleep only	1.71	(1.17, 2.50)	0.006	1.60	(1.09, 2.37)	0.018
both insomnia symptoms	3.14	(2.30, 4.30)	<0.001	5.52	(4.08, 7.48)	<0.000
Financial stability (0–4)	0.72	(0.64, 0.81)	<0.000	0.63	(0.56, 0.71)	<0.000
Healthy relationships						
no visit, no restriction	1	-	-	1	-	-
no visit, with restriction	3.45	(1.01, 11.83)	0.049	37.11	(12.70, 108.43)	<0.001
visit, with restriction	2.65	(1.61, 4.36)	<0.001	8.33	(5.10, 13.60)	<0.001
visit, no restriction	0.40	(0.30, 0.52)	<0.001	0.33	(0.24, 0.44)	<0.001
Community engagement (0–8)	0.88	(0.85, 0.92)	<0.001	0.80	(0.77, 0.84)	<0.001
Medication management						
Self-manage, no difficulty/no meds	1	-	-	1	-	-
Self-manage, difficulty	2.65	(1.90, 3.70)	<0.001	4.74	(3.34, 6.72)	<0.001
Others manage	5.23	(2.49, 10.97)	<0.001	81.13	(54.00, 193.83)	<0.001
Non-physical activities						
No	1	-	-	1	-	-
Yes	2.09	(1.76, 2.49)	<0.001	3.06	(2.56, 3.66)	<0.001
Email/text and internet						
Both email/text and internet	1	-	-	1	-	-
Either email/text or internet	1.25	(0.97, 1.61)	0.081	0.62	(0.45, 0.86)	0.003
Neither	1.63	(1.35, 1.97)	<0.001	1.29	(1.07, 1.55)	0.006

**TABLE 4:** Associations of individual self-care behavior or self-care behavior patterns at baseline and patterns of change in activities of daily living (ADLs) disability over time: Results from latent class regression model applied to each individual self-care behavior or self-care behavior pattern after adjusting for age, sex, race, and education.

Self-care behaviors or patterns	Shifting to assistance vs. maintaining independence			Shifting to accommodation/difficulty vs. maintaining independence		
	Odds Ratio	95% CI	*p*-value	Odds Ratio	95% CI	*p*-value
Favorable vs. unfavorable behavioral pattern	0.14	(0.11, 0.17)	<0.001	0.76	(0.64, 0.89)	0.001
Physical activity (walk, vigorous activities)						
Neither	1	-		1	-	
Either	0.34	(0.27, 0.42)	<0.001	0.73	(0.60, 0.88)	0.001
Both	0.10	(0.08, 0.13)	<0.001	0.50	(0.41, 0.62)	<0.001
Sleep quality						
no insomnia	1	-		1	-	
difficulty initiating sleep only	1.70	(1.30, 2.24)	<0.001	1.25	(0.99, 1.58)	0.066
difficulty maintaining sleep only	1.46	(0.98, 2.19)	0.065	1.17	(0.84, 1.63)	0.341
both insomnia symptoms	4.17	(3.07, 5.67)	<0.001	1.86	(1.39, 2.49)	<0.001
Financial stability (0–4)	0.68	(0.60, 0.76)	<0.001	0.86	(0.78, 0.96)	0.004
Healthy relationships						
no visit, no restriction	1	-		1	-	
no visit, with restriction	21.09	(8.36, 53.20)	<0.001	2.84	(1.03, 7.87)	0.045
visit, with restriction	5.84	(3.56, 9.59)	<0.001	2.21	(1.35, 3.61)	0.002
visit, no restriction	0.48	(0.36, 0.64)	<0.001	0.93	(0.71, 1.20)	0.573
Community engagement (0–8)	0.88	(0.84, 0.91)	<0.001	1.02	(0.98, 1.06)	0.259
Medication management						
Self-manage, no difficulty/no meds	1	-		1	-	
Self-manage, difficulty	4.44	(3.08, 6.40)	<0.001	2.08	(1.52, 2.86)	<0.001
Others manage	49.30	(28.70, 84.69)	<0.001	1.14	(0.56, 2.32)	0.714
Non-physical activities						
No	1	-		1	-	
Yes	2.17	(1.81, 2.60)	<0.001	1.29	(1.10, 1.52)	0.002
Email/text and internet						
Both email/text and internet	1	-		1	-	
Either email/text or internet	0.88	(0.65, 1.20)	0.435	1.30	(1.05, 1.60)	0.016
Neither	1.32	(1.10, 1.58)	0.003	1.15	(0.98, 1.36)	0.084

### Five-Year All-Cause Mortality Risk Among all Participants

**FIGURE 2:**
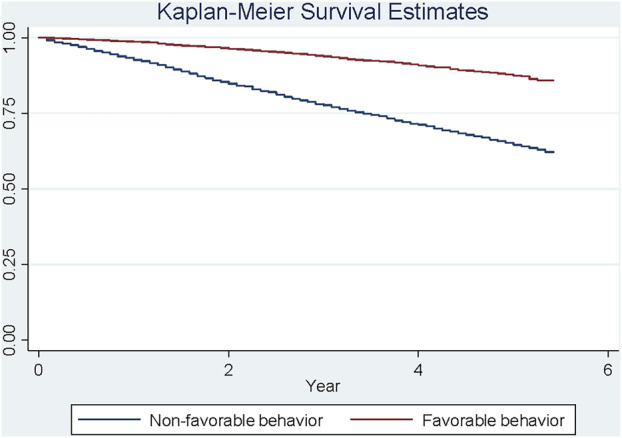
Xxxx.

Participants with an unfavorable self-care behavioral pattern at baseline died quicker than participants with a favorable pattern (Cox regression-based test: *p* < 0.001) (Figure 2). After adjusting for baseline age, education, race, and sex, a favorable pattern was associated with 61% reduction in mortality risk (Hazard Ratio = 0.39, *p* < 0.001), compared to an unfavorable pattern.

### Years of Able and Healthy Life Among Individuals Who Died

During the 5-year follow-up period, 1,575 participants died. Decedents who exhibited a favorable baseline self-care behavioral pattern were younger (mean = 80 vs. 82 years old), female (50 vs 57%), Non-Hispanic white (87 vs. 80%), and more educated (85 vs. 66% with high school diploma or higher) (Table 5). The group who had a favorable (vs. unfavorable) baseline self-care behavioral pattern lived a median of 2.9 (vs. 2.1) YoL, 1.3 (vs. 0.5) YHL, 1.5 (vs. 0.0) YAL, and 1.0 (vs. 0.0) YHAL during the 5-year follow-up period. Among decedents, those who had a favorable (vs. unfavorable) baseline self-care behavioral pattern lived 1.23 times longer YoL (95% CI:1.14–1.32), 1.91 times longer YHL (95% CI:1.68–2.18), 2.2 times longer YAL (95% CI:1.90–2.54), and 2.87 times longer YHAL (95% CI:2.38–3.46) during the 5-year follow-up period (Table 6). Women exhibiting favorable patterns had a greater advantage than men in expecting more YoL (Mean ratio = 1.25 for women vs. 1.17 for men; *p* = 0.076 for interaction) and YAL (Mean ratio = 2.39 for women vs. 2.03 for men; *p* = 0.066 for interaction) compared to counterparts with an unfavorable pattern.

**TABLE 5:** Summary of baseline characteristics by self-care behavioral patterns among those who died over the 5-year follow-up period (2011–2016)^a^.

Baseline characteristic % or mean (standard error)	Total sample (n = 1,575)	Unfavorable behavioral pattern (n = 1,177; 71.4%)	Favorable behavioral pattern (n = 398; 28.6%)	*p*-value
Age (year)	81.0 (0.24)	81.7 (0.28)	79.5 (0.46)	<0.001
Female	54.8%	56.9%	49.6%	0.025
Race				
White, non-Hispanic	81.9%	80.0%	86.5%	0.042
Black, non-Hispanic	8.6%	9.5%	6.3%	
Hispanic	5.8%	6.7%	3.7%	
Other	3.7%	3.8%	3.6%	
Education				
≤8th grade	13.6%	16.1%	7.2%	<0.001
9th-12th grade (no diploma)	15.2%	18.0%	8.3%	
High school graduate or higher	71.2%	65.9%	84.6%	
Physical activity (walk, vigorous activities)				
Neither	47.9%	63.0%	9.9%	<0.001
Either	37.7%	33.2%	49.0%	
Both	14.4%	3.8%	41.1%	
Sleep quality				
no insomnia	67.8%	60.5%	85.9%	<0.001
difficulty initiating sleep only	12.2%	15.1%	4.7%	
difficulty maintaining sleep only	6.6%	7.2%	5.3%	
both insomnia symptoms	13.4%	17.2%	4.1%	
Financial stability (0–4)	2.88 (0.03)	2.74 (0.03)	3.22 (0.04)	<0.001
Healthy relationships				
no visit, no restriction	13.7%	18.1%	2.7%	<0.001
no visit, with restriction	7.5%	10.5%	0.0%	
visit, with restriction	11.4%	14.9%	2.6%	
visit, no restriction	67.4%	56.5%	94.7%	
Community Engagement (0–8)	3.70 (0.06)	3.16 (0.06)	5.05 (0.12)	<0.001
Medication Management				
Self-manage, no difficulty/no meds	71.2%	61.9%	94.2%	<0.001
Self-manage, difficulty	6.9%	7.3%	5.8%	
Others manage	22.0%	30.8%	0%	
Non-physical activities				
Yes	46.5%	52.9%	30.5%	<0.001
Email/text and internet				
Both email/text and internet	20.7%	8.0%	47.5%	<0.001
Either email/text or internet	13.2%	11.8%	16.3%	
Neither	66.1%	80.2%	36.2%	
Years of Life^b^	2.34 (2.42)	2.09 (2.42)	2.92 (2.41)
Years of Healthy Life^b^	0.58 (1.75)	0.46 (1.21)	1.29 (2.38)	<0.001
Years of Able Life^#^	0.42 (1.63)	0 (1.08)	1.50 (2.42)	<0.001
Years of Healthy and Able Life^#^	0 (1.00)	0 (0.42)	1.00 (2.17)	<0.001

a Weighted percentages to ensure population representation.

b Median (inter-quartile range) are presented here.

**TABLE 6:** Ratio^a^ (95% confidence interval) of years of life (or healthy or able or healthy and able life) over 5-year follow-up period comparing those with favorable vs. unfavorable behavioral pattern at baseline.

Outcome	Years of Life^d^	Years of healthy Life^e^	Years of able Life^e^	Years of healthy and able Life^e^
All those who died^b^ (n = 1,545)	1.23 (1.14, 1.32)	1.91 (1.68, 2.18)	2.20 (1.90, 2.54)	2.87 (2.38, 3.46)
Men^c^ (n = 678)	1.17 (1.05, 1.30)	1.95 (1.62, 2.36)	2.03 (1.69, 2.46)	2.81 (2.20, 3.61)
Women^c^ (n = 867)	1.25 (1.13, 1.39)	1.89 (1.57, 2.27)	2.39 (1.90, 3.01)	2.91 (2.18, 3.88)

a sampling weights were incorporated in the estimation of the ratio for population representation.

b Adjusting for baseline age, education, gender, and race.

c Adjusting for baseline age, education, and race.

d From Poisson regression.

e From negative binomial regression.

## Discussion

In this study, we operationalized eight self-care behaviors that older adults can engage in to decrease their risk of losing functional independence due to disability as they age. Two distinct patterns of self-care behaviors emerged which were identified as “favorable” and “unfavorable.” In this nationally representative cohort of Medicare beneficiaries, engaging in self-care behaviors, such as physical activity, financial stability, healthy relationships, community engagement, and technology use, helped distinguished a favorable pattern from an unfavorable pattern. More specifically, a favorable self-care behavioral pattern displayed less medication management by others and fewer non-physical activities. For mobility disability outcomes, a favorable self-care behavioral pattern had a substantial reduction in shifting into the assistance class and a sizeable reduction in shifting into the class of needing accommodation or having difficulty. A similar trend emerged for the ADLs disability outcomes, except for a more modest reduction in shifting into the class of needing accommodation or having difficulty. In addition to evaluating how favorable and unfavorable patterns factor into a more independent profile, we assessed how these patterns manifest in mortality among older adults. Participants with a favorable pattern at baseline had 61% reduction in mortality than those with an unfavorable pattern over 5 years. Among participants who died during the 5 years, those with a favorable pattern of self-care behaviors had 0.6 fewer disability-years compared to those with an unfavorable pattern of self-care behaviors and this trend appeared more so in women.

Our findings emphasize that a favorable self-care behavioral pattern promotes a more independent profile among older adults, in comparison to an unfavorable self-care pattern. These results complement existing literature, which specifically address how self-care behaviors are modifiable and may influence other self-care behaviors. Jacob et al. (2016) emphasized the transformational nature of self-care behaviors in adjusting disability by demonstrating that lifestyle factors could compress or expand the disabled period, thereby supporting the compression of morbidity theory (Fiatarone et al., 1994) Existing studies, notably randomized controlled trials evaluating the impact of self-care behaviors, indicated that self-care behaviors do not occur in isolation and may influence the development of other self-care behaviors. For instance, Varma et al. (2016) found that community engagement increases physical activity, which may promote healthy relationships (Carlson et al., 2015; Varma et al., 2016) Furthermore, several self-care behaviors may yield differential magnitude of effect sizes than others (Carlson et al., 2015; Varma et al., 2016) Accordingly, we chose to assess the impact of self-care behaviors, instead of physiological risk factors, in improving disability and longevity among older adults, to inform public health interventions to encourage the uptake of modifiable favorable self-care behavioral patterns.

Strengths of this study include examining the combined effects of health-promoting self-care behaviors, which to our knowledge has not been done previously, in order to evaluate their influence on prolonging independence as one ages. Furthermore, baseline self-care behaviors remained stable over time in the NHATS cohort, allowing this measurement to be a valid proxy for follow-up periods up to 5 years. This study provides evidence of the late-lifestyle, independent of other risk factors. Those with a favorable self-care pattern promotes a more independent profile compared to those with an unfavorable pattern. A favorable self-care pattern can be applied from early adulthood to avoid disability in later life as a result of accumulated unhealthy lifestyle patterns.

Limitations may include the inability to draw causal inference. The baseline behavioral patterns may have been affected by underlying health status, such as preclinical disability. Larger representation of Black and Hispanic participants will allow for future stratified models. Assessing more comprehensive combinations of self-care behavior and new controlled-effectiveness trials in assessing the validity of self-care behavioral interventions for older adults are needed. The National Council on Aging sponsors a low-cost, 10-week self-care behavioral program, known as the Aging Mastery Program® (AMP), that addresses six of the self-care behaviors that we studied, including: physical activity, sleep, medication management, healthy relationships and community engagement. A quasi-experimental study demonstrated that AMP was an effective program that improved physical activity and advanced care planning among older adults (McCallion et al., 2016) Thus, there is a significant need for further development, implementation, evaluation, and dissemination of evidence-based self-behavioral interventions to contribute to higher degrees of independence and longevity among today’s older adults.

## Conclusion

Overall, self-care behaviors were associated with decreases in both moderate (accommodation/difficulty) and severe (assistance) disability. Second, there was a differential risk decrease depending on the self-care behavior; thus, future resources should target those with stronger effects. Third, we found that health-promoting self-care behaviors compress disability. Finally, our findings support further rigorous effectiveness trials of a comprehensive, community-based self-care behavioral program in older adults, such as the Aging Mastery Program^®^ (AMP), that was feasible to implement and scale-up, covering a broad range of health-promoting self-care behaviors.

## Data Availability

The original contributions presented in the study are included in the article/Supplementary Material, further inquiries can be directed to the corresponding author.
