# Supplementary material for: Patterns of Self-Care Behaviors and Their Influence on Maintaining Independence: The National Health and Aging Trends Study
Source: Front Aging. 2021 Dec 24;2:770476. doi: 10.3389/fragi.2021.770476 (PMC9261429; doi:10.3389/fragi.2021.770476)
Supplement: Supplementary file 1 [file DataSheet1.docx]

**eTable1. Summary of self-care behaviors and disability outcomes by study visit (NHATS 2011 – 2016)^a^**

| Variable | Visit 1  n = 7,609 | Visit 2  n = 6,469 | Visit 3  n = 5,364 | Visit 4  n = 4,443 | Visit 5  n = 3,953 | Visit 6  n = 3,531 |
| --- | --- | --- | --- | --- | --- | --- |
| Physical activity (walk, vigorous activities) |  |  |  |  |  |  |
| Neither  Either  Both | 30.5%  39.0%  30.6% | 30.0%  38.4%  31.6% | 29.3%  40.4%  30.4% | 31.2%  39.0%  29.8% | 31.5%  37.7%  30.8% | 30.8%  39.3%  29.9% |
| Sleep quality |  |  |  |  |  |  |
| no insomnia  difficulty initiating sleep only  difficulty maintaining sleep only  both insomnia symptoms | 72.9%  11.5%  5.5%  10.1% | 75.2%  10.6%  4.6%  9.5% | 75.5%  10.8%  5.6%  8.1% | 76.5%  10.6%  4.8%  8.0% | 76.4%  11.2%  4.3%  8.1% | 75.8%  10.8%  5.2%  8.2% |
| Financial stability (0-4) | 2.94 (0.01) | 2.99 (0.01) | 3.00 (0.01) | 3.05 (0.02) | 3.02 (0.02) | 3.04 (0.02) |
| Healthy relationships |  |  |  |  |  |  |
| no visit, no restriction  no visit, with restriction  visit, with restriction  visit, no restriction | 10.0%  2.5%  6.6%  80.9% | 8.5%  2.2%  6.5%  82.7% | 9.1%  2.3%  6.6%  82.0% | 9.6%  3.0%  5.7%  81.6% | 11.3%  2.5%  6.4%  79.7% | 11.7%  2.5%  5.8%  80.0% |
| Community engagement (0-8) | 4.22 (0.03) | 4.32 (0.03) | 4.33 (0.04) | 4.38 (0.04) | 4.38 (0.04) | 4.40 (0.05) |
| Medication management |  |  |  |  |  |  |
| Self-manage, no difficulty/no meds  Self-manage, difficulty  Others manage | 85.9%  7.1%  7.0% | 85.7%  6.5%  7.8% | 84.6%  7.7%  7.7% | 84.7%  8.0%  7.3% | 83.4%  9.0%  7.6% | 84.0%  9.0%  7.6% |
| Non-physical activities |  |  |  |  |  |  |
| Yes | 35.6% | 34.7% | 33.6% | 32.2% | 30.5% | 30.8% |
| Email/text and internet |  |  |  |  |  |  |
| Both email/text and internet  Either email/text or internet  Neither | 36.5%  17.6%  45.9% | 38.1%  18.9%  43.0% | 38.9%  20.0%  41.1% | 40.2%  20.1%  39.7% | 40.9%  20.7%  38.4% | 42.2%  20.0%  37.7% |
| Mobility disability |  |  |  |  |  |  |
| Fully able  Accommodate/reduced frequency  Difficulty  Need assistance | 66.8%  14.9%  4.8%  13.4% | 66.7%  12.3%  4.7%  16.4% | 64.6%  12.8%  4.6%  18.0% | 61.3%  14.4%  5.0%  19.3% | 60.7%  15.6%  5.7%  18.0% | 59.6%  15.0%  5.9%  19.5% |
| Activities of daily living (ADLs) disability |  |  |  |  |  |  |
| Fully able  Accommodate/reduced frequency  Difficulty  Need assistance | 42.5%  39.7%  4.7%  13.1% | 38.9%  40.5%  3.9%  16.7% | 35.8%  41.8%  3.9%  18.5% | 32.2%  43.0%  4.9%  19.9% | 29.8%  45.5%  4.8%  19.9% | 27.3%  46.7%  5.2%  20.9% |

^a^weighted percentages to ensure population representation

**eTable2a. Summary of self-care behaviors and disability outcomes within the “unfavorable group” by study visit (NHATS 2011 – 2016)^a^**

| Variable | Visit 1  n = 4,128 | Visit 2  n = 3,480 | Visit 3  n = 2,776 | Visit 4  n = 2,185 | Visit 5  n = 1,868 | Visit 6  n = 1,628 |
| --- | --- | --- | --- | --- | --- | --- |
| Physical activity (walk, vigorous activities) |  |  |  |  |  |  |
| Neither  Either  Both | 55.9%  39.4%  4.8% | 48.7%  40.4%  10.9% | 47.1%  40.6%  12.3% | 51.1%  39.3%  9.6% | 50.7%  36.9%  12.4% | 50.8%  38.1%  11.1% |
| Sleep quality |  |  |  |  |  |  |
| no insomnia  difficulty initiating sleep only  difficulty maintaining sleep only  both insomnia symptoms | 60.0%  15.9%  6.8%  17.3% | 66.1%  13.5%  5.5%  14.9% | 66.4%  14.2%  6.0%  13.4% | 67.3%  13.4%  5.2%  14.1% | 66.8%  14.0%  4.3%  14.9% | 66.4%  13.7%  5.5%  14.4% |
| Financial stability (0-4) | 2.66 (0.02) | 2.73 (0.02) | 2.73 (0.02) | 2.77 (0.02) | 2.77 (0.02) | 2.76 (0.03) |
| Healthy relationships |  |  |  |  |  |  |
| no visit, no restriction  no visit, with restriction  visit, with restriction  visit, no restriction | 18.4%  5.3%  12.7%  63.6% | 13.8%  4.3%  10.3%  71.6% | 14.5%  4.8%  10.0%  70.9% | 15.7%  5.8%  8.0%  70.5% | 19.1%  4.8%  9.0%  67.1% | 19.5%  4.6%  8.8%  67.1% |
| Community engagement (0-8) | 3.09 (0.03) | 3.28 (0.04) | 3.33 (0.05) | 3.33 (0.05) | 3.31 (0.06) | 3.30 (0.06) |
| Medication management |  |  |  |  |  |  |
| Self-manage, no difficulty/no meds  Self-manage, difficulty  Others manage | 76.9%  8.2%  14.8% | 77.6%  6.8%  15.6% | 76.1%  8.4%  15.5% | 77.7%  7.8%  14.5% | 76.0%  9.5%  14.5% | 77.0%  9.2%  13.8% |
| Non-physical activities |  |  |  |  |  |  |
| Yes | 47.9% | 47.6% | 46.5% | 44.4% | 41.7% | 43.9% |
| Email/text and internet |  |  |  |  |  |  |
| Both email/text and internet  Either email/text or internet  Neither | 8.9%  15.9%  75.2% | 12.0%  16.5%  71.6% | 12.2%  19.7%  68.2% | 12.0%  19.0%  69.0% | 12.1%  21.6%  66.3% | 12.4%  21.4%  66.1% |
| Mobility disability |  |  |  |  |  |  |
| Fully able  Accommodate/reduced frequency  Difficulty  Need assistance | 47.8%  19.4%  7.8%  25.0% | 48.6%  15.8%  7.2%  28.4% | 45.6%  17.0%  7.0%  30.4% | 42.5%  17.4%  6.6%  33.5% | 42.2%  18.9%  8.1%  30.9% | 41.4%  18.3%  8.6%  31.6% |
| Activities of daily living (ADLs) disability |  |  |  |  |  |  |
| Fully able  Accommodate/reduced frequency  Difficulty  Need assistance | 34.0%  36.6%  6.6%  22.7% | 29.5%  36.5%  5.3%  28.7% | 25.5%  37.5%  5.4%  31.6% | 22.9%  38.2%  5.7%  33.2% | 21.5%  40.1%  6.1%  32.3% | 20.6%  39.5%  6.7%  33.2% |

^a^weighted percentages to ensure population representation

**eTable2b. Summary of self-care behaviors and disability outcomes within the “favorable group” by study visit (NHATS 2011 – 2016)^a^**

| Variable | Visit 1  n = 3,481 | Visit 2  n = 2,989 | Visit 3  n = 2,588 | Visit 4  n = 2,258 | Visit 5  n = 2,085 | Visit 6  n = 1,903 |
| --- | --- | --- | --- | --- | --- | --- |
| Physical activity (walk, vigorous activities) |  |  |  |  |  |  |
| Neither  Either  Both | 7.7%  38.6%  53.8% | 15.0%  36.7%  48.3% | 16.1%  40.2%  43.7% | 17.7%  38.8%  43.4% | 19.1%  38.2%  42.8% | 18.6%  40.0%  41.4% |
| Sleep quality |  |  |  |  |  |  |
| no insomnia  difficulty initiating sleep only  difficulty maintaining sleep only  both insomnia symptoms | 84.5%  7.5%  4.3%  3.7% | 82.5%  8.3%  3.9%  5.2% | 82.1%  8.4%  5.3%  4.2% | 82.7%  8.8%  4.5%  4.0% | 82.5%  9.4%  4.3%  3.7% | 81.5%  9.0%  5.1%  4.4% |
| Financial stability (0-4) | 3.19 (0.01) | 3.20 (0.02) | 3.20 (0.02) | 3.24 (0.02) | 3.18 (0.02) | 3.22 (0.02) |
| Healthy relationships |  |  |  |  |  |  |
| no visit, no restriction  no visit, with restriction  visit, with restriction  visit, no restriction | 2.5%  0.0%  1.0%  96.4% | 4.3%  0.5%  3.5%  91.7% | 5.1%  0.5%  4.2%  90.2% | 5.5%  1.2%  4.1%  89.2% | 6.3%  1.0%  4.8%  87.9% | 6.9%  1.2%  4.0%  88.0% |
| Community engagement (0-8) | 5.23 (0.04) | 5.16 (0.04) | 5.06 (0.05) | 5.10 (0.05) | 5.06 (0.05) | 5.06 (0.06) |
| Medication management |  |  |  |  |  |  |
| Self-manage, no difficulty/no meds  Self-manage, difficulty  Others manage | 93.9%  6.1%  0.0% | 92.1%  6.3%  1.6% | 90.8%  7.3%  2.0% | 89.5%  8.1%  2.4% | 88.3%  8.6%  3.1% | 88.2%  8.4%  3.4% |
| Non-physical activities |  |  |  |  |  |  |
| Yes | 24.6% | 24.2% | 24.0% | 23.8% | 23.2% | 22.9% |
| Email/text and internet |  |  |  |  |  |  |
| Both email/text and internet  Either email/text or internet  Neither | 57.1%  18.9%  24.0% | 55.4%  20.4%  24.2% | 54.8%  20.2%  25.0% | 55.8%  20.8%  23.5% | 56.3%  20.3%  23.4% | 57.5%  19.3%  23.2% |
| Mobility disability |  |  |  |  |  |  |
| Fully able  Accommodate/reduced frequency  Difficulty  Need assistance | 83.9%  10.9%  2.2%  3.1% | 82.0%  9.3%  2.5%  6.2% | 79.3%  9.6%  2.7%  8.4% | 74.8%  12.2%  3.9%  9.1% | 73.3%  13.4%  4.1%  9.2% | 71.3%  12.8%  4.2%  11.7% |
| Activities of daily living (ADLs) disability |  |  |  |  |  |  |
| Fully able  Accommodate/reduced frequency  Difficulty  Need assistance | 50.1%  42.5%  2.9%  4.5% | 47.0%  43.9%  2.7%  6.4% | 44.0%  45.1%  2.7%  8.2% | 38.9%  46.5%  4.4%  10.3% | 35.4%  49.1%  4.0%  11.5% | 31.5%  51.3%  4.2%  12.9% |

^a^weighted percentages to ensure population representation

**eTable3. Selecting number of classes in latent class analysis.**

| **Outcome** | **Number of Classes** | **AIC** | **BIC** | **n-adjusted BIC** | **LMR-LRT** | **Entropy** |
| --- | --- | --- | --- | --- | --- | --- |
| Self-care behaviors at baseline | 2 | 117492 | 117721 | 117616 | P<0.01 | 0.580 |
|  | 3 | 117121 | 117461 | 117305 | P=0.440 | 0.520 |
| Mobility disability over time | 2 | 51623 | 51879 | 51762 | P<0.01 | 0.810 |
|  | 3 | 49259 | 49648 | 49470 | P<0.01 | 0.773 |
|  | 4 | 48507 | 49027 | 48789 | P=0.822 | 0.726 |
| Activities of daily living (ADLs) disability over time | 2 | 63032 | 63288 | 63171 | P<0.01 | 0.784 |
|  | 3 | 57305 | 57693 | 57515 | P<0.01 | 0.775 |
|  | 4 | 56538 | 57058 | 56820 | P=0.779 | 0.726 |

AIC: Akaike information criterion; BIC: Bayesian information criterion; LMR-LRT: Lo-Mendell-Rubin adjusted likelihood ratio test

**eTable4. Prevalence of positive response to each response category by latent trajectory profile: Results from 3-class latent class model (Class 1: maintaining independence over time; Class 2: shifting to accommodation or difficulty; Class 3: shifting to assistance).**

| **Prevalence (%)** | **Mobility** | | | **Activities of daily living (ADLs)** | | |
| --- | --- | --- | --- | --- | --- | --- |
|  | Class 1 | Class 2 | Class 3 | Class 1 | Class 2 | Class 3 |
| Visit 1 |  |  |  |  |  |  |
| Fully able | 92.0% | 43.1% | 10.6% | 85.9% | 18.0% | 11.7% |
| Accommodate/reduced frequency | 5.9% | 40.0% | 15.0% | 12.5% | 72.1% | 26.5% |
| Difficulty | 0.5% | 10.6% | 12.7% | 0.3% | 6.5% | 9.0% |
| Need assistance | 1.6% | 6.3% | 61.7% | 1.3% | 3.4% | 52.7% |
| Visit 2 |  |  |  |  |  |  |
| Fully able | 94.4% | 39.3% | 5.4% | 86.9% | 11.4% | 7.3% |
| Accommodate/reduced frequency | 3.3% | 39.3% | 10.0% | 11.5% | 78.6% | 18.3% |
| Difficulty | 0.6% | 12.4% | 8.9% | 0.3% | 6.3% | 5.8% |
| Need assistance | 1.7% | 9.0% | 75.7% | 1.3% | 3.8% | 68.7% |
| Visit 3 |  |  |  |  |  |  |
| Fully able | 93.1% | 29.4% | 1.8% | 83.1% | 7.8% | 4.5% |
| Accommodate/reduced frequency | 3.8% | 42.3% | 6.7% | 13.7% | 80.8% | 10.4% |
| Difficulty | 0.3% | 14.3% | 7.7% | 0.5% | 6.5% | 4.8% |
| Need assistance | 2.8% | 14.1% | 83.8% | 2.7% | 4.9% | 80.2% |
| Visit 4 |  |  |  |  |  |  |
| Fully able | 91.1% | 13.3% | 2.4% | 75.4% | 6.4% | 1.0% |
| Accommodate/reduced frequency | 5.2% | 48.1% | 2.1% | 18.8% | 79.0% | 4.6% |
| Difficulty | 0.6% | 17.1% | 6.0% | 1.5% | 8.0% | 4.9% |
| Need assistance | 3.1% | 21.6% | 89.5% | 4.4% | 6.6% | 89.5% |
| Visit 5 |  |  |  |  |  |  |
| Fully able | 88.3% | 10.1% | 1.7% | 70.4% | 3.7% | 0.7% |
| Accommodate/reduced frequency | 7.4% | 47.4% | 2.6% | 24.2% | 77.1% | 5.5% |
| Difficulty | 1.2% | 19.1% | 5.6% | 0.9% | 8.8% | 3.3% |
| Need assistance | 3.1% | 23.4% | 90.1% | 4.5% | 10.4% | 90.6% |
| Visit 6 |  |  |  |  |  |  |
| Fully able | 84.3% | 10.3% | 1.3% | 62.0% | 3.9% | 0.4% |
| Accommodate/reduced frequency | 8.6% | 40.0% | 4.2% | 28.9% | 74.7% | 4.8% |
| Difficulty | 1.3% | 20.6% | 5.6% | 2.0% | 8.7% | 2.7% |
| Need assistance | 5.8% | 29.1% | 89.0% | 7.1% | 12.8% | 92.1% |

**eTable5a. Prevalence of positive response to each response category by self-care behavioral patterns: Results from 2-class latent class model.**

| **Self-care Behavior**  **% or mean (standard error)** | **Unfavorable** | **Favorable** |
| --- | --- | --- |
| Physical activity (Walk, vigorous activities) |  |  |
| Neither  Either  Both | 51.7%  40.8%  7.5% | 10.6%  37.2%  52.2% |
| Sleep quality |  |  |
| no insomnia  difficulty initiating sleep only  difficulty maintaining sleep only  both insomnia symptoms | 61.2%  15.5%  6.6%  16.6% | 83.9%  7.7%  4.4%  4.0% |
| Financial stability (0-4) | 2.69 (0.02) | 3.17 (0.02) |
| Healthy relationships |  |  |
| no visit, no restriction  no visit, with restriction  visit, with restriction  visit, no restriction | 17.3%  5.2%  12.0%  65.5% | 3.2%  0.1%  1.4%  95.3% |
| Community engagement (0-8) | 3.21 (0.05) | 5.16 (0.07) |
| Medication management |  |  |
| Self-manage, no difficulty/no meds  Self-manage, difficulty  Others manage | 77.4%  8.1%  14.5% | 93.8%  6.1%  0.0% |
| Non-physical activities |  |  |
| No  Yes | 53.4%  46.6% | 74.6%  25.4% |
| Internet/email use |  |  |
| Both email/text and internet  Either email/text or internet  Neither | 12.1%  16.5%  71.5% | 55.7%  18.5%  25.8% |

**eTable5b. Prevalence of positive response to each response category by self-care behavioral patterns: Results from 3-class latent class model.**

| **Self-care Behavior**  **% or mean (standard error)** | **Unfavorable** | **Less Favorable** | **Favorable** |
| --- | --- | --- | --- |
| Physical activity (Walk, vigorous activities) |  |  |  |
| Neither  Either  Both | 66.0%  29.5%  4.5% | 32.6%  49.8%  17.6% | 10.4%  33.9%  55.7% |
| Sleep quality |  |  |  |
| no insomnia  difficulty initiating sleep only  difficulty maintaining sleep only  both insomnia symptoms | 50.8%  19.3%  6.9%  23.0% | 73.1%  11.2%  6.1%  9.6% | 84.1%  7.7%  4.2%  4.0% |
| Financial stability (0-4) | 2.64 (0.05) | 2.82 (0.13) | 3.20 (0.02) |
| Healthy relationships |  |  |  |
| no visit, no restriction  no visit, with restriction  visit, with restriction  visit, no restriction | 12.6%  11.5%  24.4%  51.5% | 17.8%  0.1%  1.4%  80.1% | 1.6%  0.2%  2.1%  96.1% |
| Community engagement (0-8) | 3.46 (0.26) | 3.05 (0.13) | 5.67 (0.47) |
| Medication management |  |  |  |
| Self-manage, no difficulty/no meds  Self-manage, difficulty  Others manage | 59.8%  11.3%  28.9% | 93.0%  4.9%  2.1% | 92.8%  6.9%  0.3% |
| Non-physical activities |  |  |  |
| No  Yes | 44.8%  55.2% | 65.0%  35.0% | 73.8%  26.2% |
| Internet/email use |  |  |  |
| Both email/text and internet  Either email/text or internet  Neither | 10.0%  13.2%  76.7% | 19.5%  21.2%  59.3% | 60.8%  16.5%  22.8% |

**eTable6a. Results from 2 and 3-class LCA of physical activity over time.**

| 2-Class LCA | 3-Class LCA |
| --- | --- |
|  |  |

**eTable6b. Results from 2 and 3-Class LCA of sleep quality over time.**

| 2-Class LCA | 3-Class LCA |
| --- | --- |
|  |  |

**eTable6c. Results from 2, 3-Class LCA of healthy relationships over time.**

| 2-Class LCA | 3-Class LCA |
| --- | --- |
|  |  |

**eTable6d. Results from 2, 3-Class LCA of email/text and internet use over time.**

| 2-Class LCA | 3-Class LCA |
| --- | --- |
|  |  |

**eTable6e. Results from 2, 3-Class LCA of medication management over time.**

| 2-Class LCA | 3-Class LCA |
| --- | --- |
|  |  |

**eTable6f. Results from 2, 3-Class LCA of non-physical activities over time.**

| 2-Class LCA | 3-Class LCA |
| --- | --- |
|  |  |

**eTable6g. Results from 2, 3-Class latent profile analysis of financial stability over time.**

**Two-Class Model**

Two-Tailed

Mean S.E. Est./S.E. P-Value

Latent Class 1

Means

Visit1 2.047 0.029 70.063 0.000

Visit2 2.001 0.032 62.084 0.000

Visit3 1.933 0.032 60.533 0.000

Visit4 1.981 0.033 60.592 0.000

Visit5 1.994 0.034 58.204 0.000

Visit6 2.053 0.040 51.607 0.000

Latent Class 2

Means

Visit1 3.218 0.011 284.795 0.000

Visit2 3.283 0.012 277.694 0.000

Visit3 3.312 0.013 248.121 0.000

Visit4 3.350 0.014 234.642 0.000

Visit5 3.303 0.015 226.448 0.000

Visit6 3.316 0.014 230.186 0.000

**Three-Class Model**

Latent Class 1

Means

Visit1 1.905 0.031 61.063 0.000

Visit2 1.833 0.036 51.492 0.000

Visit3 1.778 0.033 54.569 0.000

Visit4 1.845 0.036 50.860 0.000

Visit5 1.861 0.038 48.501 0.000

Visit6 1.901 0.042 45.718 0.000

Latent Class 2

Means

Visit1 2.940 0.015 196.021 0.000

Visit2 2.973 0.015 196.932 0.000

Visit3 2.950 0.017 170.213 0.000

Visit4 2.971 0.019 154.957 0.000

Visit5 2.962 0.019 159.200 0.000

Visit6 3.001 0.018 164.951 0.000

Latent Class 3

Means

Visit1 3.556 0.019 188.060 0.000

Visit2 3.671 0.019 193.878 0.000

Visit3 3.744 0.019 196.766 0.000

Visit4 3.784 0.017 221.496 0.000

Visit5 3.681 0.020 185.061 0.000

Visit6 3.668 0.021 174.733 0.000

**eTable6h. Results from 2, 3-Class latent profile analysis of community engagement over time.**

**Two-Class Model**

Mean S.E. Est./S.E. P-Value

Latent Class 1

Means

Visit1 2.992 0.043 69.977 0.000

Visit2 2.963 0.046 64.268 0.000

Visit3 2.934 0.050 58.183 0.000

Visit4 2.913 0.056 52.305 0.000

Visit5 2.902 0.058 50.227 0.000

Visit6 2.941 0.061 48.477 0.000

Latent Class 2

Means

Visit1 6.106 0.060 102.296 0.000

Visit2 6.240 0.061 102.478 0.000

Visit3 6.164 0.061 100.318 0.000

Visit4 6.211 0.064 97.609 0.000

Visit5 6.130 0.067 92.047 0.000

Visit6 6.005 0.071 85.175 0.000

**Three-Class Model**

Latent Class 1

Means

Visit1 2.228 0.048 46.460 0.000

Visit2 2.118 0.054 39.417 0.000

Visit3 2.060 0.054 38.107 0.000

Visit4 2.034 0.057 35.630 0.000

Visit5 1.991 0.059 33.751 0.000

Visit6 2.040 0.067 30.364 0.000

Latent Class 2

Means

Visit1 6.733 0.054 125.098 0.000

Visit2 6.871 0.047 145.643 0.000

Visit3 6.781 0.052 130.680 0.000

Visit4 6.802 0.054 126.535 0.000

Visit5 6.772 0.059 113.852 0.000

Visit6 6.590 0.065 101.170 0.000

Latent Class 3

Means

Visit1 4.314 0.055 79.039 0.000

Visit2 4.377 0.055 79.567 0.000

Visit3 4.330 0.056 77.444 0.000

Visit4 4.322 0.062 70.129 0.000

Visit5 4.259 0.059 72.638 0.000

Visit6 4.231 0.060 70.588 0.000

**eTable7. Frequency distribution of YoL, YAL, YHL, and YHAL observed during the 5-year follow-up among the 1,575 who died.**

| A   | C   |
| --- | --- |
| B   | D   |
